# A Rare Variant of Guillain-Barre Syndrome Following Ad26.COV2.S Vaccination

**DOI:** 10.7759/cureus.18153

**Published:** 2021-09-21

**Authors:** Zachary P Morehouse, Amanda Paulus, Sri A Jasti, Xue Bing

**Affiliations:** 1 Family and Community Medicine, Michigan State University College of Osteopathic Medicine, East Lansing, USA; 2 Research and Development, Omni International Inc., Kennesaw, USA; 3 Internal Medicine, Michigan State University College of Osteopathic Medicine, East Lansing, USA; 4 Internal Medicine, St. Joseph Mercy Ann Arbor Hospital, Ypsilanti, USA; 5 Pulmonary and Critical Care Medicine, St. Joseph Mercy Ann Arbor Hospital, Ypsilanti, USA

**Keywords:** ad26.cov2.s vaccine, vaccine adverse reactions, vaccine adverse events, adenovirus vaccine, autoimmune, guillain-barre syndrome (gbs), covid-19 vaccine complication, covid-19 vaccine, covid-19

## Abstract

Efforts to combat the global pandemic caused by severe acute respiratory syndrome coronavirus 2 (SARS-CoV-2) range from adequate diagnostic testing and contract tracing to vaccination for the prevention of coronavirus disease 2019 (COVID-19). In the United States alone, three vaccinations have been authorized for emergency use (EUA) or approved to prevent COVID-19. The Ad26.COV2.S vaccine by Johnson and Johnson (New Brunswick, New Jersey) is the only adenovirus-based vaccine and deemed relatively effective and safe by the US Food and Drug Administration (FDA) following its clinical trial. Since its introduction, the US FDA has placed a warning on the vaccine adverse event reporting system (VAERS) after more than 100 cases of Guillain-Barre Syndrome (GBS) were reported. Herein, we outline the hospital course of a generally healthy 49-year-old female who experienced an axonal form of GBS nine days after receiving the Ad26.COV2.S vaccine.

## Introduction

Guillain-Barre Syndrome (GBS) is an acute autoimmune complication of the peripheral nervous system, mostly idiopathic, often associated with preceding infections, or occasionally, different vaccinations [1-2]. Autoreactive antibodies trigger an immune response against the host myelin or axon, leading to inflammatory polyneuropathy [1]. Clinically, patients can present with progressive ascending paresthesia, flaccid paralysis, areflexia, dysautonomia, and/or respiratory failure. Most patients have their peak symptoms two to four weeks following symptom onset [1]. The annual incidence of GBS is 0.4-4.0 cases per 100,000 population among all ages [1,3-4]. There are several variants of GBS characterized by their initial symptom presentation and progressive patterns, including acute inflammatory demyelinating polyneuropathy (AIDP), Miller Fisher syndrome, and acute motor and sensory axonal neuropathy (AMSAN) [1,5].

In July 2021, the US Food and Drug Administration (FDA) announced that Ad26.COV2.S vaccine by Johnson and Johnson had the potential linkage to GBS in a small number of patients. Herein, we describe a rare variant of GBS with a temporal relation to the Ad26.COV2.S to further the body of literature regarding this phenomenon.

## Case presentation

A 49-year-old Caucasian female with a history of neurofibromatosis type I, hypothyroidism, and multiple vitamin deficiencies presented to her primary care physician with new-onset numbness in her extremities for a duration of one day. She experienced reduced tactile sensation and numbness in her right upper extremity, extending from the elbow into her hand and bilateral lower extremities from her knees through her feet. She had associated headache with pain to palpation across her forehead, lightheadedness, and aching lumbar pain of four days duration. Her history was negative for any recent illnesses, changes to medications or lifestyle, domestic or international travel, or exposure to any known sick contacts. The patient reported receiving the Ad26.COV2.S vaccine five days prior to the onset of her symptoms.

Due to acute neurological involvement of her multiple extremities with reduced tactile sensation, the patient was directed immediately to the emergency department for further evaluation.

**Figure 1:**
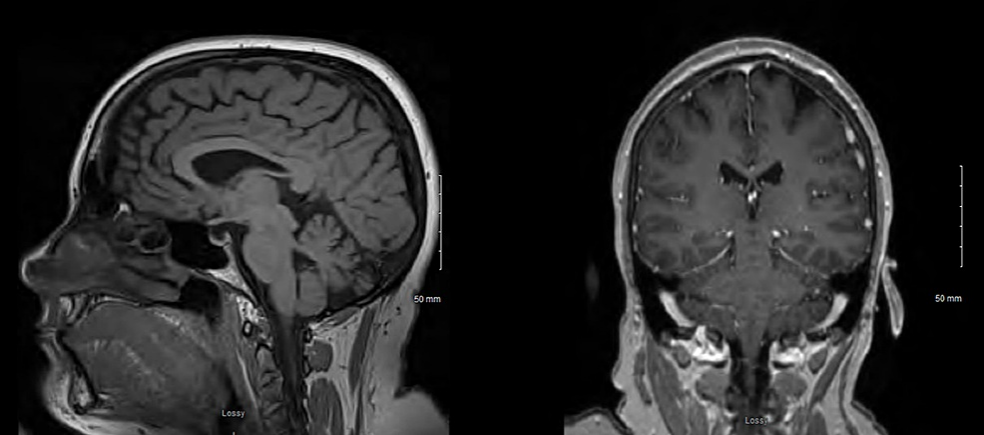
MRI brain results from initial emergency department visit demonstrating small punctate foci inconsistent with multiple sclerosis or acute neurologic sequelae and no evidence of cervical spine demyelination

During the initial emergency department evaluation, the patient endorsed difficulty walking and holding objects in addition to the previously reported symptoms. These difficulties were thought to be secondary to altered sensation rather than resulting from musculoskeletal weakness. Physical exam revealed orbital, jaw, and neck pain to palpation. The patient was afebrile, severe acute respiratory syndrome coronavirus 2 (SARS-CoV-2) negative, and showed no signs of infection or electrolyte abnormality on initial complete blood count (CBC), comprehensive metabolic panel (CMP), and urinalysis (UA). Thyroid-stimulating hormone, free thyroxine, and muscle-specific kinase antibodies were within normal limits. The only abnormal blood work seen was an equivocal B12 level of 187 ng/L (normal 180-914 ng/L). She also underwent an MRI of her brain and cervical spine for concerns of multiple sclerosis given her multiple neurologic symptoms. Brain MRI revealed small punctate foci inconsistent with multiple sclerosis or acute neurologic findings (Figure 1). Due to equivocal B12 levels and non-specific MRI findings, the patient was given a B12 injection and discharged to her home with instructions for outpatient follow-up with neurology.

The next day, she returned to the emergency department with worsening generalized weakness and paresthesia. The patient also noted new hoarseness and dysphagia, thoracic back pain, and urinary incontinence that had all developed within the 16 hours since her emergency department (ED) discharge.

In ED triage, the patient collapsed to the floor without loss of consciousness or seizure activities. She was afebrile, hypertensive to 171/98, and hypoxic with oxygen saturation in the mid-80s. Her physical exam was positive for symmetric weakness in bilateral lower extremities. A CT angiogram of the chest was ordered for concerns of a developing aortic dissection, which was unremarkable. Additional imaging was then ordered, with an MRI of the brain, which showed moderate cervical stenosis. MRI of the thoracic and lumbar spine was also conducted but was otherwise unremarkable. Her lumbar puncture revealed elevated albumin (normal <27.0 mg/dL) and IgG (normal <8.1 mg/dL) without signs of meningitis or multiple sclerosis (Table 1). The patient was admitted to the medical intensive care unit (MICU) due to her rapidly progressing neurological symptoms.

**Table 1:** Lumbar puncture results from the second emergency department visit The results showed elevated albumin and immunoglobulin G (IgG). Elevated RBCs were likely attributed to trauma during the procedure, given the normal range for WBC, glucose, and proteins.

CSF Values		
	Sample	Reference
WBC	0	<5 lymphocytes/mcL
RBC	368	0-10 RBC/mcL
Glucose	77	40-80 mg/dL
Protein	58	15-60 mg/dL
Albumin	46	0.0-27.0 mg/dL
IgG	8.8	0.0-8.1 mg/dL
Oligoclonal Bands	Negative	Negative

Neurology evaluated the patient the next day, which was 12 days since she received her SARS-CoV-2 vaccine. Her physical examination was notable for bilateral extraocular muscles weakness, decreased pinprick sensation in all four extremities, with preserved temperature and vibratory sensation, 4 out of 5 strength in the right upper extremity, bilateral proximal lower extremities, and bilateral finger flexion, with preserved 5 out of 5 strength in the left upper extremity. Additionally, reflexes were tested and showed 1+ in bilateral biceps and brachioradialis, and absent at the bilateral patella and Achilles.

By hospital day 4, the patient became almost quadriplegic, along with bilateral facial nerve paralysis and a weak gag reflex. She required bilevel positive airway pressure (BiPAP) initially and eventually intubation. Given her rapid neurological decompensation, with sensory symptoms with negative workup and recent history of vaccination, clinically, neurology suspected this was a rare variant of GBS called acute motor and sensory axonal neuropathy (AMSAN). She was started on intravenous immunoglobulin (IVIG).

On hospital day 13, she remained intubated without significant improvement despite four days of IVIG. She developed symptomatic bradycardia that required pacemaker placement. On hospital days 23-32, the patient received a course of plasmapheresis, due to lack of clinical improvement, and a tracheostomy. On hospital days 33-54, the patient underwent physical rehab and ventilatory weaning at Select Hospital. On hospital days 54-63, the patient was readmitted by neurology for a second round of plasmapheresis and an additional dose of IVIG. Following these treatments, she had gradual improvement in her weakness. On hospital day 64, the patient was transferred back to Select Hospital.

## Discussion

GBS is an acute and idiopathic autoimmune disease of the peripheral nervous system, which has been reported to be temporally linked to many viral infections, medications, and vaccinations [1]. This disease process often presents with progressive and symmetrical motor loss, starting in the extremities in a stocking and glove pattern [3]. However, the presentation can vary as seen in this case. Multiple variants of GBS have been categorized by their clinical presentation, but no mechanistic differences in autoimmune nerve damage have been identified with these variants to date [1,5].

Variants of GBS are categorized by their symptom presentation and progression patterns [1,5]. Many variants of GBS have been categorized based on their clinical course, including acute inflammatory demyelinating polyneuropathy (AIDP), acute motor and sensory axonal neuropathy (AMSAN), Miller-Fisher Syndrome (MFS), and multifocal motor neuropathy (MMN) [5]. Initially, the neurologists on the treatment team believed our patient likely had AIDP, the most common subtype of GBS in the United States. This was due to the rapidly ascending nature of her paralysis, autonomic neuropathy, cranial nerve deficits, and pain. However, AIDP does not typically include sensory deficits as seen as the first symptom experienced by this patient. MFS was also on the differential due to the involvement of the patient’s extraocular muscles and cranial nerves with concerns for pending ophthalmoplegia [1,5-6]. However, our patient was ultimately diagnosed with AMSAN given her ascending paralysis in addition to sensory deficits, a unique feature associated with this particular subtype of GBS [5-6].

GBS is mostly a clinical diagnosis, as we currently do not have any definitive diagnostic tests for either confirmation of the disease or determination of its inciting cause [1]. New or changing medications, such as fluoroquinolone antibiotics, recent infectious diseases, including campylobacter or influenza infections, and vaccinations, such as the shingles vaccine, have all been reported in the literature as potential inciting events for GBS cases [1,3,7]. Electrodiagnostic studies, such as electromyography (EMG), can assist in distinguishing subtypes of GBS by classifying the disease as demyelinating or axonal [1,7]. However, at the early stages of the disease process, patients may not meet the criteria for neurophysiologic evaluation so clinical judgment is heavily relied upon for initial diagnosis and initiation of management.

Treatment of GBS is plasmapheresis and/or IVIG with supportive care regardless of its subtypes [7]. Comparatively, there is no substantial documented difference in efficacy between either plasmapheresis or IVIG treatment for GBS patients [7]. The rapid initiation of treatment for these patients is critical in reducing the risk for morbidity and mortality. Therefore, determining the specific variation of GBS is of lower importance than simply having a high clinical index of suspicion for GBS in general and beginning prompt treatment [5,7].

The temporal relations documented in the literature showing a connection between an inciting agent and GBS rely almost exclusively on a diagnosis of exclusion [1,7-9].

In July 2021, the US FDA issued a warning on the potential association of GBS with Johnson and Johnson’s Ad26.COV2.S vaccination (New Brunswick, New Jersey) against SARS-CoV-2 after more than 100 cases of GBS had been reported. Previously both adenovirus and adenovirus vector vaccine-associated GBS were reported in the medical literature. Therefore, it is reasonable to anticipate similar potential adverse reactions following current SARS-CoV-2 vaccination [10-12]. Further investigation and monitoring of these patients will be a valuable tool to improve treatment and predict prognosis.

## Conclusions

Our patient presented with a rare variant of GBS following the administration of a novel vaccine with no known connection to GBS at the time of presentation in the Spring of 2021. This case highlights the importance of considering atypical presentations of a relatively known disease. Additionally, this was a complex case with a therapeutic dilemma. Due to its variable clinical presentation and lack of specific diagnostic testing, it is critical that medical providers keep a high index of suspicion for this disease process. As described in our patient, the unique presentation of the rare variant of GBS, AMSAN, can make a prompt diagnosis challenging and difficult. When evaluating patients with progressive paralysis with and without neuropathy, GBS should remain in the differential, even if patients do not follow exactly the characteristic stocking and glove pattern. Early involvement of the neurology team in suspected cases may help diagnose patients presenting with some of the unique neurological features, ultimately allowing for quicker intervention.

It is imperative to obtain a thorough history, looking for any potential inciting event to strengthen clinical suspicion of a GBS diagnosis. It has become even more paramount during this current pandemic with the wide use of vaccinations. Given the unique presentation of this patient's GBS potentially associated with SARS-CoV-2 vaccination and the rapidly developing literature surrounding the progression of the COVID-19 pandemic and global vaccination campaigns, additional studies on similar cases are warranted. Further classification of additional cases following similar patterns may help elucidate potential mechanisms of action for this phenomenon, as well as guide quicker recognition and treatment algorithms.
